# Consortium on Automated Analytical Laboratory Systems (CAALS)

**DOI:** 10.1155/S1463924690000141

**Published:** 1990

**Authors:** H. M. (Skip) Kingston

**Affiliations:** Inorganic Analytical Research Division Center for Analytical Chemistry National Institute of Standards and Technology Gaithersburg Maryland 20899 USA


Journal of Automatic Chemistry, Vol. 12, No. 3 (May–June 1990), pp. 113–115

### Consortium on Automated Analytical Laboratory Systems (CAALS)

**H. M. (Skip) Kingston**

*Inorganic Analytical Research Division, Center for Analytical Chemistry, National Institute of Standards and Technology, Gaithersburg, Maryland 20899, USA*

Industry and government both depend heavily on analytical laboratory measurements. Although these measurements provide critical feedback in many situations, such as product quality assurance, acceptance testing, and regulatory compliance, they are generally viewed with mixed feelings. On the positive side, industry recognizes that improvements in product and process capability often depend on advancements in analytical measurement technologies. A firm's competitiveness depends in part on the accuracy, sensitivity, reliability, and controllability of its measurements and processes. The precision and control are reflected in the quality and cost of the firm's products. On the negative side, analytical measurements are expensive because they are labour intensive, requiring high-cost personnel and expensive equipment.

Scientists at the National Institute of Standards and Technology (NIST) project that during the next decade analytical measurement capabilities will experience technical growth equal to that of the previous three decades combined. Furthermore, NIST perceives that analytical measurement tasks are ripe for automation and that development of an automated analytical laboratory offers US industry opportunities for significant improvements in the quality and cost of analytical operations. To accelerate development of these benefits and to provide a broad utilization base, NIST has proposed the establishment of an industry-government research Consortium on Automated Analytical Laboratory Systems (CAALS). The purpose of the consortium is to accelerate the advancement of automated analytical systems, to improve efficiency and data quality, and to promote transferability of analytical methods so that US-based industry enjoys a competitive advantage in chemical measurement technology.

It has taken approximately 9 months to form the consortium with the assistance of many industry leaders who have had significant input into the structure and proposed directions of CAALS. A committee composed of members from the Center for Analytical Chemistry and other related areas of NIST was established to guide the formation of the consortium.

### Why a consortium?

Cannot each firm solve these problems as it sees fit? In NIST's view, a consortium format seems best for several reasons.

### *Generic benefits*

The consortium solves a major problem for all participating firms. Variants of the same solutions need not be reinvented separately by each company via in-house programmes. Needless duplication of research is avoided.

### *Standardized common interfaces*

Solutions once developed for many are more likely to be adopted as a standard for all. These solutions can provide design standards for instrument and software developers. Through technology, software, and other technical solutions, the consortium may be better able to develop common bases for instrument and software development. This standardization will help users, because they will find their equipment more compatible.

### *Affordability*

Several large US and international organizations, such as NIST, have recognized the impact of automated analytical measurement systems on their competitiveness and have implemented internal programs to automate portions of their activities. However, most firms cannot afford to implement such extensive in-house programmes. A consortium allows small and middle sized businesses to become involved in chemical automation research and to enjoy the benefits of a research programme of this scope at modest cost. Participants from large firms benefit by leaving the generic projects to the consortium's programme and by using in-house talents to pursue projects of interest to the company.

### *Broader talent pool*

The consortium will draw its talent from member companies nationwide, from other interested government agencies, and from NIST (which has the greatest diversity of analytical chemists in the world). Only qualified top talent will be encouraged (or accepted) to be part of the consortium's project teams. In addition, the consortium will develop a pool of qualified graduate students who can fill voids in employment needs in this topic area. The result will be a group of individuals whose talents offer more diversity than could be assembled by a single firm.

### *Greater product breadth*

The consortium will pursue automation technologies in many different areas of chemical analysis with the goal of developing automated laboratory systems. Through the consortium, it will be possible to pursue more projects simultaneously than could be pursued through the efforts of a single corporation.

© American Chemical Society 1990.

*Relevant participants*

The consortium format is conducive to involving diverse, but relevant, perspectives of various groups with vested interests in the consortium's research. These groups include users, instrumentation and software developers, regulatory agencies, and professional standard-setting organizations.

### Consortium philosophy

The basic philosophy of this consortium is one of flexibility and shared decision-making to evolve guidelines and functions through which the industrial community and NIST work as partners to develop automated chemical analysis technologies. NIST believes that working shoulder to shoulder' enriches everyone involved in the research effort. Active industrial involvement focuses the research on industrial needs and accelerates the transfer of technology to member firms.

This philosophy of active involvement is reflected in the consortium design. The more a consortium member contributes to the active research of the consortium, the more benefits that member accrues. Members of the NIST consortium will share responsibility for setting the research agenda and research priorities; collaborating actively in the development of consortium technologies; sharing the intellectual property (publications and patents) developed by the consortium; and accessing and participating in research seminars, workshops, and training sessions on consortium activities and products.

### The technical programme

Technical guidelines have been developed to focus the efforts of the consortium. These guidelines define the scope of the consortium's research efforts within which specific projects will occur; the specifics of the research directions will be dependent on the desires of the consortium members.

By definition, an automated analytical laboratory system includes two main components (see table): sample preparation, which is divided into analyte release and analyte separation components; and an analyte detection or identification and quantification component.

Analyte detection components have been successfully automated over the years. Until recently, automation efforts neglected sample preparation, and errors in sample preparation were identified as a major cause of inaccurate chemical analysis. The CAALS research programme will address all aspects of automating the chemical analysis system but will emphasize sample preparation. The technical programme of the consortium will centre on establishing definitive criteria for selecting chemical analysis components that are amenable to automation; optimizing the measurement quality of each chemical analysis system; and providing user-oriented computer programs for system selection, optimization, and control. Careful consideration will be given to the selection of the proper components for automation. Once promising components are selected, significant research and development effort will be expended to assure that they can be used effectively as components of an automated system. Examples of each of the three categories are shown in the table.

Once a set of components has been chosen, a substantial amount of additional research and development will be done to optimize the performance of each of the components with respect to the automation. In general, the concept of modular design in all aspects of the components and system operation will be followed. All hardware and software components will be modular. To the fullest extent possible, these components will be made interchangeable and compatible. The concept currently used in object programming will also be used in the hardware design to simplify the assembly of components into a system. Computer control will be implemented in a hierarchical mode in which the lowest set of software modules will interact with specific components while a higher set of computer software modules (system software) will interact with the lower set to monitor and control the overall operation of the automated chemical analysis system. Expert systems will be devised to assist the chemist in selecting the proper chemical analysis system, providing information for the implementation of recommended procedures, and evaluating analysis results.

The consortium will provide a useful service to its members by choosing practical, ubiquitous modular laboratory components for applications and testing of automated methodology. The first phase in the logical development of an automated chemical analysis system is to automate each of the selected components individually, while keeping in mind the ultimate goal of a fully automated chemical analysis system. During this development, members of the consortium will be able to use

*Chemical analysis systems.*

| Analyte release | Analyte separation | Analyte detection |
| --- | --- | --- |
| Microwave dissolution | Liquid chromatography | ICP-OES elemental detection |
| Fusion | Gas chromatography | Atomic absorption |
| Solvation | Matrix modification | ICP/MS elemental detection |
| Solvent extraction | Liquid-liquid extraction | Flame ionization detection |
| Supercritical fluid extraction | | Spectroscopic detection |
| Solid-phase extraction | | Mass spectrometry |

the individual components in their laboratories well in advance of the complete systems. The components and systems will be replicated in the laboratories of members who wish to use the system. This phase could involve collaboration with the instrument manufacturers.

During the development of an analytical system, research efforts will also be directed toward advanced integration of automated systems. To accomplish this, researchers must adhere to the following guidelines.

*Build chemical analysis expertise*

To provide a chemical analysis involving a specific material, set of analytes and analysis error, it is necessary for any given analytical system to develop specific operational protocols. These protocols generate the data necessary to achieve intelligent instrument control. With the appropriate computer software, the system will be 'smart' as well as automated, Expert systems and other forms of artificial intelligence will be used to play appropriate roles in guiding the various operations of the chemical analysis.

*Develop standard reference methods*

These activities are necessary to replace common manual techniques, to establish their validity, and to aid in their implementation. Implementation of these automated standard methods will be accomplished with the active involvement of voluntary standards organizations, professional societies and regulatory government agencies.

*Build in quality assurance*

The automated analytical system will be developed so that it will monitor and adjust the procedural conditions to provide a controlled environment for high-quality chemical analysis. These methods can be used to ensure reproducibility and transferability of the results between laboratories.

### CAALS workshop

On 28 September 1989, NIST held a workshop to describe CAALS to representatives of industry, government agencies, and universities. On the first day of the workshop, NIST scientists discussed areas of current expertise and described research programs that support automation efforts in inorganic, organic, gas and particulate analysis in the Center for Analytical Chemistry. These areas include broad expertise in chemical analysis; access to experts in associated fields such as robotics, statistical engineering, and artificial intelligence; expertise in quality assurance, including the certification of reference materials; ongoing commitment to data analysis; and continuing interactions with regulatory government agencies and industry.

In addition, direct benefits to consortium members were described. These include the ability to leverage costly R&D to accelerate the development of automated systems, promote collaboration between analytical instrument users and their manufacturers, participate in any patent and copyrights generated within the consortium, and affect long-term savings by manifesting the concept of standard interfaces, modular design, and versatility in design of the fundamental building blocks of an automated chemical analysis system. Although it will be the responsibility of the consortium members in collaboration with NIST staff to set the research agenda, NIST scientists presented their concept of the first automated system for inorganic trace element analysis consisting of microwave dissolution, liquid chromatographic separation, and ICP-optical emission detection.

On the second day of the workshop, after a review of the important discussions and conclusions from the previous day, the first organizational meeting of the consortium was held. Prospective members took part in a spirited 2 hour discussion of the research agenda, and a number of suggestions were made about the organic automated system. An extraction procedure coupled to a chromatographic separation was suggested, and it was observed that an important aspect of these analyses is the minimization of toxic waste. It was also suggested that appropriate prototype projects relate to some EPA-mandated procedure. It was agreed that more input is needed to arrive at a demonstration project that best suits the needs of CAALS members, and suggestions were received from prospective members through November 1989.

The concept of modular design and interconnectivity of subcomponents was discussed. Standardization of interfaces was a high priority, and it was decided that the subcomponents should have 'open architecture' to allow for expansion and interchangeability.

At this meeting and the subsequent one at the 1990 Pittsburgh conference, charter members were accepted to CAALS; potential CAALS members should now contact NIST for further information.†

Other members of the CAALS Committee who contributed their unique expertise to the development of this consortium include Harry Hertz, Director, Center for Analytical Chemistry (CAC); Barry Diamondstone, Deputy Director, CAC; James R. DeVoe, Chief, Inorganic Analytical Research Division (IARD); W. E. May, Chief, Organic Analytical Research Division (OARD); Rance A. Velapoldi, Chief, Gas and Particulate Science Division; M. R. Rubin, General Counsel, NIST; W. F. Koch, Deputy Chief, IARD; Bruce Mattson, Manager, Technology Commercialization, Office of Research and Technology Applications, NIST; R. L. Watters, Jr., Supervisory Research Chemist, IARD; W. A. MacCrehan, Research Chemist, OARD; Neomy Soffer, Guest Scientist, CAC; R. M. Colton, Robert M. Colton Associates, Consultant; and R. D. Kilmer, Center for Manufacturing Engineering, NIST.

### Acknowledgement

Reprinted with permission from *Analytical Chemistry* **1989**, *61*, 1381–1384A. Copyright 1989 American Chemical Society.†

† Dated information has been altered to reflect the latest events relevant to the Consortium and two figures from the original article have been omitted.

